# The Role of Thrombophilia in Pregnancy

**DOI:** 10.1155/2013/516420

**Published:** 2013-12-18

**Authors:** Elisabeth M. Battinelli, Ariela Marshall, Jean M. Connors

**Affiliations:** ^1^Brigham and Women's Hospital, Division of Hematology, 75 Francis Street, Boston, MA 02115, USA; ^2^Dana-Farber Cancer Institute, Department of Hematology and Medical Oncology, 450 Brookline Avenue, Smith 353, Boston, MA 02115, USA

## Abstract

Thrombotic disease is a major cause of peripartum morbidity and mortality worldwide. Development of thrombosis in pregnancy is multifactorial due to the physiologic changes of pregnancy—which induce a relative hypercoagulable state—as well as physical changes leading to increased stasis and also the effects of both the inherited and the acquired thrombophilias. In this review, we discuss the impact of each of these factors on the development of thrombosis as well as the evidence for the impact of pregnancy-associated thrombosis on pregnancy outcome. We then discuss the use of both prophylactic and therapeutic anticoagulation during pregnancy and the puerperium. We review the indications and dosing recommendations for administration of anticoagulation in a context of discussing the evidence including the lack of evidence and formal guidelines in this area. We briefly address the role of the new oral anticoagulants in pregnancy and conclude that significant further research in women with thrombophilias and pregnancy-associated thrombosis may help clarify the management of this condition in the future.

## 1. Introduction

The risk of venous thromboembolic events (VTE) is high during pregnancy due to both physiologic changes of pregnancy and the additional impact of the inherited and acquired thrombophilias. The overall rate of venous thromboembolic events in pregnancy is 200 per 100,000 deliveries [1]. The main risk appears to occur in the postpartum period where the incidence increases almost 2.5-fold and is estimated at 500 per 100,000. The majority of these events are deep vein thrombosis as opposed to the more deadly pulmonary embolism. Venous thromboembolic events remain a leading cause of death which has been estimated to range from 1.2 to 4.7 per 100,000 pregnancies.

Inherited and acquired thrombophilias contribute further to an increased predisposition to thrombotic events. The overall impact of the inherited and acquired thrombophilias is low in the nonpregnant population, and the majority of patients never experience a thrombotic event. During pregnancy, however, the increased risk of thromboses in patients with inherited and acquired thrombophilias can be substantial and warrants consideration, especially as thrombosis is the leading cause of mortality during pregnancy. Fifty percent of the patients with thrombosis during pregnancy will be found to have an underlying thrombophilia.

## 2. Pathophysiologic Changes during Pregnancy

The physiological changes that occur during pregnancy are mainly responsible for the increased thrombogenicity of the peripartum period. A number of clotting factors including factor VII, factor VIII, Factor X, von Willebrand factor, and fibrinogen are elevated as a result of hormonal changes. At the same time, resistance to activated protein C increases in the second and third trimesters and the activity of protein S is decreased due to changes in the total protein S antigen level [2]. There is also an increase in a number of inhibitors of the fibrinolytic pathway such as activatable fibrinolytic inhibitor (TAFI) and plasminogen activator inhibitor 1 and 2 (PAI-1 and PAI-2) [3, 4].

In addition, the physical changes of pregnancy result in an increased thrombotic state. Increased pressure on the pelvic veins from the gravid uterus and decreased flow in the lower extremities result in increased stasis. Relative compression of the left iliac vein by the right iliac artery as it courses across the vessel leads to an increase of clots in the left iliac vein [5, 6]. Although stasis increases throughout the course of pregnancy and leg pain and swelling are more frequent during the third trimester, incidence of DVT is distributed relatively equally across trimesters [7].

Concomitant diseases such as systemic lupus erythematous or sickle cell disease as well as other risk factors including obesity, decreased mobility, increased age, and smoking all increase the risk of thrombosis. It has been estimated that the women who are over 35 and pregnant have a 1.38-fold increased risk of having a clotting event during the peripartum period [8]. Women who have had spontaneous clotting events in the past have an increased risk of developing a second event with an estimated rate of recurrence of 10.9% during pregnancy [9].

Overall, both the physiologic and anatomic changes of pregnancy take several weeks to resolve after delivery, and the risk of thrombosis remains elevated (and indeed even elevated compared to pregnancy) until approximately 6 weeks postpartum [1].

## 3. Acquired and Inherited Thrombophilias

### 3.1. Acquired Disorders

The main acquired thrombophilia leading to increased risk of VTE in pregnancy is the antiphospholipid antibody syndrome. A number of criteria must be met in order to make a diagnosis of antiphospholipid antibody syndrome. This includes one or more episodes of document thrombosis, and/or recurrent (3 or more) early miscarriages occurring in the first 10 weeks of gestation, 1 or more fetal losses occurring after 10 weeks or preterm delivery at ≤34 weeks for preeclampsia or placental insufficiency. These clinical scenarios must also be accompanied by defined laboratory criteria. Lupus anticoagulant (LAC) must be present in the plasma on 2 separate occasions at least 12 weeks apart and/or anticardiolipin (aCL) antibody of either the IgG or IgM isotype (or both) present in plasma at medium to high titers (>40), or the presence of anti-beta2-glycoprotein (anti-b2GPI) of IgG or IgM isotype must be present on 2 or more occasions again at least 12 weeks apart [10].

Antiphospholipid antibody syndrome has been associated with an odds ratio of 15.8 for clotting risk during pregnancy [8]. There is a clear association between antiphospholipid antibodies and pregnancy loss [11]. The persistence of anticardiolipins and lupus anticoagulant is strongly associated with increased risk of pregnancy-related thrombotic complications yet the management of these patients is not well defined [12].

### 3.2. Inherited Disorders

Inherited thrombophilias are present in over 50% of the cases of pregnancy-associated VTE. There are a number of inherited disorders which lead to an increased thrombotic risk (Table 1).

This includes both point mutations as well as deficiencies in anticoagulant proteins. The most frequent abnormalities are Factor V Leiden mutation and the prothrombin gene mutation. These mutations occur in 2–5% of the Caucasian population, accounting for the main genetic abnormalities associated with VTE [13–15]. The Factor V Leiden mutation is caused by the substitution of arginine by glutamine at amino acid position 506. This results in a conformational change in the protein that contributes to activated protein C resistance through disrupting factor Va inactivation. The prothrombin 20210 mutation results from the substitution of guanine by adenine in the 20210 noncoding position, leading to an increase in the level of plasma prothrombin that is likely from increased stability of prothrombin mRNA. 44% of the pregnancy-associated clots in patients with a history of VTE are associated with Factor V Leiden mutations [16]. The prevalence of the Prothrombin G20210A mutation is 17% in patients who develop VTE during pregnancy [17].

The risk of pregnancy-associated VTE in these disorders has been assessed in a recent meta-analysis which involved the review of 9 studies [18]. The risk of homozygous Factor V Leiden for thrombosis is associated with an odds ratio of 43.4 while homozygous prothrombin mutation is associated with 24.4 odds ratio. Heterozygosity for Factor V Leiden is associated with a 8.3 odds ratio while heterozygosity for prothrombin G20210A is associated with an odds ratio of 6.8. More simply, it has been estimated that 1 in 500 Factor V Leiden heterozygotes and 1 in 200 prothrombin G20210A heterozygotes will experience a thrombotic event during pregnancy [19].

Deficiencies in normal coagulation proteins can also lead to a hypercoagulable state. Abnormalities in protein S, protein C, and antithrombin are all associated with thrombophilia during pregnancy. As discussed earlier, changes in these coagulation factors occur as a physiological manifestation of pregnancy. Deficiencies in these coagulation factors lead to a more profound change in coagulation levels. The odds ratio for VTE occurrence in pregnancy is 4.8 for women with protein C deficiency, 3.2 for protein S deficiency, and 4.7 for antithrombin deficiency [18]. The risk for a thrombotic event during pregnancy for women with protein C deficiency is 1 in 113, 1 in 42 for antithrombin deficiency type 2, and 1 in 3 for antithrombin deficiency type 1 [19].

The association of thrombophilia with mutations in MTHFR is controversial. The C667T mutation in MTHFR gene results in a higher level of homocysteine which is essential for metabolizing vitamin B12 and folate. As a natural physiological consequence of pregnancy, homocysteine levels can be low [20]. Although previously it was postulated that there was an association, it has recently been shown that the presence of homozygous mutations in the MRHFR gene are not significantly associated with increased risk of VTE during pregnancy (odds ratio 0.7) [21].

It can therefore be concluded that all inherited thrombophilias except for the MTHFR mutation are associated with increased risk for VTE during pregnancy (Table 2). Based on these statistics, it appears that the greatest risk occurs in those homozygous for Factor V Leiden or prothrombin 20210 mutations, Factor V Leiden and prothrombin G20210A compoundheterozygotes, and those with antithrombin deficiency.

## 4. Outcomes Related to Thrombophilias in Pregnancy

The contribution of thrombophilias to adverse outcomes in pregnancy is controversial. Studies tend to be small, have population selection bias, and have differences in diagnostic criteria. There are, however, a number of disorders that have been associated with thrombophilia including preeclampsia, placental abruption, intrauterine growth delay, and fetal loss.

### 4.1. Pregnancy Loss in Pregnant Women with Inherited Thrombophilias

Many studies have tried to address this issue and there is still controversy regarding the importance of thrombophilia in fetal loss. Association is often difficult to uncover because of inherent study design issues in the pregnant population. Studies examining the role of Factor V Leiden and the prothrombin 20210 gene mutations are not conclusive, with some studies suggesting an important role in incidence of fetal loss and others being less clear [22, 23]. Another study which included over 5000 women found that there was indeed a strong association between Factor V Leiden and risk of stillbirth with an odds ratio of 10.9 [24]. In this study, there was not a similar association established for early fetal loss, and the prothrombin gene mutation was not associated with any increased risk. In a smaller study which involved only 100 pregnant women, there was again an association between Factor V Leiden and stillbirth but also prothrombin gene mutation. In this study, however, only late pregnancy loss and not early pregnancy loss was associated with these mutations [25]. In the NOHA (Nimes Obstetricians and Hematologists) study which was based on a cohort of over 32,000 patients in a case-control design, of the 18% of the patients who experienced pregnancy loss, there was a clear association between heterozygosity for Factor V Leiden with an odds ratio of 3.46 and prothrombin gene mutations with an odds ratio of 2.60 [26]. These losses mainly occurred after the tenth week of pregnancy with no associations found in early pregnancy. Based on these studies, it does appear that there is an association between Factor V Leiden and still birth, but the association is small as it was revealed by a prospective cohort study which showed that the risk was low at 4.2% compared to 3.2% for noncarriers [27].

Two meta-analyses demonstrated that the presence of the Factor V Leiden or prothrombin 20210 gene mutation was associated with increased risk of pregnancy loss in the first or second trimester as well as with recurrent pregnancy losses [18, 28]. The role of the other thrombophilias is less clear; the meta-analysis by Rey and colleagues found that protein C and antithrombin deficiency were not linked with fetal loss, whereas protein S deficiency was associated with late term fetal loss.

The most conclusive prospective controlled study examining multiple causes of thrombophilia and their relationship to fetal loss is the EPCOT (European Prospective Cohort on Thrombophilia) study, which evaluated 843 women with thrombophilia including 571 women with 1524 pregnancies compared with 541 control women, 395 of whom had 1019 pregnancies [29]. The rate of fetal loss was higher in those women who had more than one thrombophilia with an odds ratio of 14.3 for stillborn births. The association for women with one thrombophilic condition was 29% versus 23% in the control group with an odds ratio of 1.35. All of the thrombophilias had a trend towards increased risk of still birth or late fetal loss. The odds ratio for stillbirth for individual defects were antithrombin deficiency of 5.2, protein C of 2.3, protein S deficiency of 3.3, and Factor V Leiden 2.0. There was not however convincing evidence of a link between thrombophilias and miscarriage earlier in pregnancy with only a suggestion that antithrombin deficiency may play a role.

Another cohort study of over 490 patients found that there was no association between maternal thrombophilia and early pregnancy loss [30]. In fact, the authors suggested that perhaps there is a protective advantage to thrombophilia for survival of early pregnancies with a lower rate of recurrent losses. However, the study did find a modest association with adverse pregnancy outcomes including late fetal loss or still birth after 14 weeks of gestation. Overall, these studies would suggest that having an underlying thrombophilia is associated with a late pregnancy loss or a stillbirth but not an increased risk of early pregnancy loss.

### 4.2. Thrombophilia and Placental Abruption

Placental abruption has also been associated with an underlying thrombophilic condition in the pregnant patient although a consistent association has not been established. This was recently supported by Roqué and colleagues who looked at a number of adverse placental outcomes in women with thrombophilia [30]. In this study it was found that the risk of abruption increased as the number of thrombophilic conditions carried by the patient increased in a dose dependent manner. The most significant association between abruption and thrombophilia was established for patients with antithrombin deficiency. Other studies have also looked at this risk but have not revealed as clear an association. Other studies have suggested that hyperhomocystinemia, but not the MTHFR mutation, may be associated with placental abruption in both a cohort and meta-analysis approach [31, 32]. Therefore, it appears that antithrombin deficiency and hyperhomocystinemia may increase the risk of placental abruption but statistical significance is lacking.

### 4.3. Thrombophilia and Preeclampsia

Another placental pathology that has been associated with thrombophilia is preeclampsia, and it has been estimated that 40% of the patients who present with preeclampsia harbor an underlying thrombophilia [33, 34]. As with the other adverse pregnancy outcomes, however, the data is still mixed. The cases associated with thrombophilia appear to have a severe phenotype with an increased risk of HELLP (hypertension, elevated liver function tests, and low platelets). Studies that have looked at these associations are difficult to interpret because of statistical analysis issues including between-study heterogeneity. A large meta-analysis found that Factor V Leiden was associated with preeclampsia with an odds ratio of 2.5 for severe hypertension during pregnancy [35]. Other studies however have not provided such convincing evidence, with only a small increased risk attributed to Factor V Leiden in preeclampsia [36–38]. Similar results were found in the case of the prothrombin gene mutation, with only a small risk of preeclampsia associated with this hypercoagulable state [39–41]. Other thrombophilias may also be associated with preeclampsia as suggested by a recent meta-analysis which found a 12.7 odds ratio for an association with protein S deficiency and a 21.5 odds ratio for an association with protein C [31]. Unfortunately, the analysis included many small studies which weakened the statistical analysis overall and no definitive conclusions can be drawn.

Others have also addressed whether there is a role for thrombophilias in adverse pregnancy outcomes that results in low birth weight babies or intrauterine growth delay. A meta-analysis that looked at the roles of Factor V Leiden and prothrombin gene mutation as well as MTHFR homozygosis and the risk of intrauterine growth restriction did not reveal an underlying association [25]. Another meta-analysis did find an association between protein S deficiency and fetal growth delay with an odds ratio of 10.2 [31]. Again, these analyses are not definitive because of the small sample size of the component studies included and the wide confidence intervals which diminish the impact of the findings.

Although these studies have been inclusive in many of the associations between thrombophilias and poor pregnancy outcomes, many still base treatment decisions on these minimally conclusive statistical data. Because of these associations, some have even suggested that inherited thrombophilias should be routinely tested in the general population. This is unlikely, however, to yield important therapeutic benefits as these conditions are very rare in the general population and even less likely to be associated with modifiable adverse pregnancy outcomes. The most recent guidelines from the American College of Obstetrics and Gynecology postulate that women in whom knowledge of a thrombophilia will directly impact clinical management should be considered for screening [42]. Therefore, women with a history of spontaneous VTE or first degree relatives with significant clotting histories should be considered for testing. ACOG guidelines do not recommend screening women with a history of recurrent or nonrecurrent early fetal losses or adverse pregnancy outcomes due to lack of evidence from clinical data. Screening is controversial in women who experience the loss in later stages of pregnancy and who have placental pathology that suggests that ischemia, infarct, or vessel thrombosis may have contributed to the fetal demise since there is a low rate of recurrence of these outcomes and the clinical data is still lacking.

All testing for these thrombophilic disorders should occur well out from clotting events or pregnancy as acute issues can affect testing results. Also, the patients should not be receiving anticoagulation when testing is performed as antithrombin levels can be inaccurate in patients using heparin products and protein C and protein S levels will be lower in patients on warfarin.

## 5. Administration of Anticoagulation during Pregnancy

The anticoagulant of choice during pregnancy is low molecular weight heparin (LMWH), although adjusted-dose unfractionated heparin (UHF) can also be used. Low molecular weight heparin is preferred because of its extended half-life, better bioavailability, and ease of use and decreased incidence of bone loss in comparison to unfractionated heparin. Benzyl alcohol is frequently used as a preservative for multidose vials of unfractionated heparin, and when administered to neonates can result in respiratory distress and even death. Unfractionated heparin preserved with benzyl alcohol should be used cautiously immediately prior to delivery. For patients with acute VTE on treatment dose heparin, admission to the hospital for intravenous unfractionated heparin prior to delivery may be warranted. The low volume of prophylactic dose unfractionated heparin administered, or the use of prefilled syringes which do not contain benzyl alcohol, is less concerning. Warfarin is usually avoided after the first trimester because of concern for warfarin embryopathy. Anticoagulation with low molecular weight heparin is usually initiated during the antepartum period and switched at the 36th week of pregnancy to unfractionated heparin in order to avoid concern for epidural anesthesia complications that can occur with the longer half-life of low molecular weight heparins. Anticoagulation for VTE should be continued for at least 3–6 months from development of VTE but if VTE is found early in gestation, anticoagulation should be continued through delivery and for at least 4–6 weeks postpartum depending on recovery from birth and underlying thrombophilic conditions. In the postpartum period, either continuation of low molecular weight heparin or bridging to warfarin is acceptable options.

The optimal prophylactic dose of heparin or LMWH has not been determined for pregnant women. Pregnant women have been shown to require higher doses of UFH to achieve both prophylactic and therapeutic levels of anticoagulation [44]. Therapeutic dose LMWH requires dose adjustment during pregnancy as weight increases. Peak anti-Xa activity has been found to be lower in pregnant women than postpartum women [45]. While many mechanisms, such as increased renal clearance, increased plasma volume, and increased procoagulant protein levels, are thought to play a role in the need for increased heparin or LMWH dose, it is difficult to perform studies in pregnant women. Use of intermediate dose UFH or LMWH is an accepted strategy for VTE prophylaxis in pregnant women with increased risk of recurrent VTE, and is endorsed by ACCP guidelines [46].

For patients with moderate to high risk of recurrent VTE (prior DVT and strong thrombophilia), intermediate intensity anticoagulation is recommended. Intermediate intensity dosing can consist of 40 mg enoxaparin every 12 hours or enoxaparin 1 mg/kg once daily. Suggested anticoagulant doses are in Table 3.

Target anti-Xa level for prophylactic dose LMWH is 0.1–0.3 four hours after administration. For those who require therapeutic dose, weight-based dosing of either enoxaparin (1 mg/kg every 12 hours) or dalteparin (100 U/kg every 12 hours) can be used, with a goal antifactor Xa level of 0.6–1.0 four hours after administration [47]. Unfractionated heparin can be dose-adjusted to achieve a PTT from1.5 to 2.5 times the control parameters.

While there is significant interest in the new oral anticoagulants (the direct factor Xa inhibitors rivaroxaban and apixaban and the direct thrombin inhibitor dabigatran), only rivaroxaban has been FDA approved for treatment of venous thromboembolism, and as of yet there is no data to support their use during pregnancy. Pregnant women were excluded from the trials which led to approval of these agents for cardiovascular and thrombotic indications. The new oral anticoagulants are not included in the ACOG recommendations for treatment of venous thromboembolism in pregnancy, and are recommended against during both pregnancy and breast-feeding on the basis of grade 1C evidence in the 2012 ACCP guidelines.

## 6. Risk-Based Modeling for Anticoagulation

While patients who develop VTE during their pregnancy should be treated in a similar manner regardless of whether or not they have an underlying thrombophilia, patients with an inherited or acquired thrombophilia but without acute clot do not necessarily merit anticoagulation. A number of studies have tried to risk-stratify the importance of thrombophilia in a pregnant patient's overall clotting risk. A prospective study assigned pregnant women with a confirmed history of thrombophilia or a past VTE to different levels of anticoagulation using a score based on history of VTE, type of thrombophilia, age, BMI, multiparous birth, and immobilization [48]. Low risk patients received no anticoagulation prior to delivery, intermediate risk patients were started on LMWH in the third trimester, and high risk patients were anticoagulated from time of enrollment in the study. The rate of VTE was very low, with only 3 deep vein thrombosis occurring in the entire study. This trial supports the use of risk assessment in clinical management of patients with thrombosis during pregnancy. Another trial addressed withholding anticoagulation in pregnant patients with prior history of VTE [49]. The rate of thrombotic events was low even in patients with a prior history of VTE who did not receive anticoagulation in the antepartum period. The main criticism of this trial, however, is that it enrolled a small numbers of patients with history of inherited thrombophilia. Finally, a meta-analysis was performed to address this issue which included almost 65 studies and a total of 2777 pregnancies in which anticoagulation was administered successfully both for prophylaxis and also treatment [50].

As a potential guideline for how to stratify patients with thrombophilia during pregnancy, Fogerty and Connors developed a risk category assessment to aid in clinical management [43]. The table of risk categories is shown (Table 4).

## 7. Prophylactic Anticoagulation during Pregnancy

While it is clear that the association between early pregnancy loss and thrombophilia does not warrant the generalized use of anticoagulation, the use of anticoagulation for women with a history of fetal loss later in pregnancy is controversial [30]. A number of studies have addressed the use of anticoagulation in pregnancy in patients with a history of thrombophilia and adverse pregnancy outcomes. One trial of 160 women with a history of fetal loss after 10 weeks of gestation and the presence of a hypercoagulable state including Factor V Leiden, prothrombin gene mutation, or protein S deficiency randomized women to either low dose aspirin or prophylactic doses of enoxaparin for the duration of pregnancy [51]. Women in the enoxaparin group had 86% live births in comparison to a rate of 28% in those who received aspirin. This trial is not without faults, however, as the birth rate in the aspirin arm was much lower than expected, and the study was also not blinded. A benefit was also seen in a cohort study of women with protein C deficiency, protein S deficiency, or antithrombin deficiency [52]. The use of thromboprophylaxis during pregnancy resulted in a significantly lower rate of fetal loss (0% versus 45%), but this study is also difficult to interpret since it was small and not randomized or blinded. Another study found that women with thrombophilia and a history of first pregnancy loss were able to deliver without any adverse outcomes in their next pregnancy in the absence of anticoagulation [53]. Thus, just as the data establishing the relationship between thrombophilia and adverse pregnancy outcomes is confusing, so too is the impact of anticoagulation to correct the underlying hypercoagulable tendency.

The use of anticoagulation has not shown significant benefit in women with history of pregnancy loss but without known thrombophilia [54–56]. A systemic review of randomized controlled trials looking at the use of low molecular weight heparin in women with recurrent or late nonrecurrent fetal loss with no history thrombophilia was inconclusive with no discernable benefit of anticoagulation in this group [57]. Clearly more studies are needed in women both with and without thrombophilias in the setting of recurrent pregnancy loss.

Anticoagulation for known antiphospholipid syndrome is far more straightforward as it is associated with a striking impact on pregnancy outcome. One trial of women with positive antiphospholipid antibodies and recurrent pregnancy loss showed significantly improved outcomes with the use of both aspirin and heparin compared to aspirin alone, with 71% live births in those women receiving combined therapy versus 42% live births in those who received aspirin alone [58]. Another study similarly showed a 50% increased rate of live birth with combination therapy [59]. This issue is not however without controversy, as it was shown in a trial in which similar rates of live births were seen in women with antiphospholipid syndrome treated with heparin and aspirin or aspirin alone, suggesting no additional benefit from heparin [60].

In order to develop guidelines for management of women with thrombophilia and adverse pregnancy outcomes, the American College of Chest Physicians has established recommended treatment guidelines based on both family and personal history of VTE [46]. These guidelines state that women with a history of antiphospholipid antibody syndrome based on laboratory values and previous history of pregnancy loss should receive prophylactic anticoagulation with both prophylactic dose low molecular weight heparin and low-dose aspirin (or prophylactic or intermediate dose unfractionated heparin alone). Those with known homozygosity for Factor V Leiden or prothrombin 20210 gene mutation and a positive family history of VTE are suggested to undergo antepartum and postpartum prophylaxis with prophylactic or intermediate dose low molecular weight heparin or warfarin, whereas those with homozygous mutations and no family history of VTE are suggested to undergo only postpartum prophylaxis for 6 weeks. Women with all other known thrombophilias—whether or not they have a family history of VTE—are recommended to undergo close monitoring only. Postpartum prophylaxis with prophylactic or intermediate dose low molecular weight heparin or warfarin is suggested for pregnant women with prior *personal *history of VTE.

Based on all the evidence presented, it is clear that further studies are needed to address the issues around the role of anticoagulation in preventing further pregnancy loss. While further information is gathered, clinical decisions should be based on evaluation of each case.

## 8. Conclusions

Pregnancy is a prothrombotic state in the multifactorial setting of underlying pathophysiologic changes leading to an increase of procoagulant factors, physical changes leading to increased stasis, and the additional contribution in some cases of inherited and acquired thrombophilias. The management of thrombophilia in the setting of pregnancy remains controversial. Anticoagulation may provide benefit for women both as prophylaxis and as treatment for venous thromboembolism during pregnancy. While all women with VTE should receive systemic treatment, the evidence supporting prophylactic anticoagulation is less clear. Prophylactic anticoagulation should be addressed on a case-by-case basis taking into account the inherited and acquired thrombophilias and history of prior pregnancies and their outcomes. Women with inherited thrombophilias should be counseled that although their condition may increase the risk of adverse outcomes in pregnancy, the associations are not clear and that there are no definitive studies linking the use of anticoagulation to pregnancy success in this setting. Women with acquired thrombophilia are more likely to benefit from anticoagulation and should be anticoagulated according to published guidelines.

## Figures and Tables

**Table 1:** Risk of VTE in inherited thrombophilia.

Thrombophilia	OR general population	Annual incidence of first VTE (%)	OR in pregnancy (95% CI)
AT deficiency	28.2	1.77	4.69 (1.30–16.96)
Protein C deficiency	24.1	1.52	4.76 (2.15–10.57)
Protein S deficiency	30.6	1.90	3.19 (1.48–6.86)
Factor V Leiden	7.5	0.49	Homozygous 34.4 (9.86–120.0) Heterozygous 8.32 (5.44–12.70)
Prothrombin gene mutation	5.2	0.34	Homozygous 26.36 (1.24–559.2) Heterozygous 6.80 (2.46–19.77)

CI: confidence interval.

**Table 2:** Incidence of pregnancy-associated VTE with inherited thrombophilia.

Thrombophilia	Pregnancy (%/pregnancy)	Overall (%/year)
Factor V Leiden heterozygous	2.1 (0.7–4.9)	0.5 (0.1–1.3)
Prothrombin gene mutation heterozygous	2.3 (0.8–5.3)	0.4 (0.1–1.1)
ATIII, protein C, or protein S deficiency	4.1 (1.7–8.3)	1.5 (0.7–2.8)

**Table 3:** 

	Prophylactic	Intermediate	Therapeutic
Unfractionated heparin	5000 U twice daily	10,000 U twice daily	Titrate to PTT 1.5–2.5* control
Enoxaparin	40 mg daily	40 mg every 12 hours or 1 mg/kg daily	1 mg/kg every 12 hours
Dalteparin	5000 U daily	5000 U every 12 hours	100 U/kg every 12 hours

**Table 4:** Recommendations for inherited thrombophilia based on assigned risk category.

	High	Intermediate	Low
Type of thrombophilia	Factor V Leiden homozygous, prothrombin gene homozygous, Compound heterozygous, Antithrombin deficiency, any thrombophilia + history of VTE	Low-risk thrombophilia with a strong family history of VTE	Factor V Leiden heterozygous, prothrombin gene mutation heterozygous, protein C or S deficiency, no personal/family history of VTE
Management	Intermediate or therapeutic low molecular weight heparin antepartum and for 4–6 weeks postpartum	Prophylactic dosing of low molecular weight heparin antepartum and 4–6 weeks postpartum	Clinical surveillance antepartum and anticoagulation for 4–6 weeks postpartum

Adapted from Fogerty and Connors, 2009 [43].
